# Combination therapy with tobevibart and elebsiran potently reduces hepatitis B virus surface antigen levels in preclinical *in vivo* models

**DOI:** 10.1128/aac.01127-25

**Published:** 2026-01-26

**Authors:** Julia Noack, Yesseinia Anglero-Rodriguez, Jonathan Gall, Jiayi Zhou, Sarah LeBlanc, Abigail Liebow, Anna Bakardjiev, Christy M. Hebner, Lisa A. Purcell, Vasant Jadhav, Davide Corti, Florian A. Lempp

**Affiliations:** 1 Vir Biotechnology 630422 , San Francisco, California, USA; 2 Alnylam Pharmaceuticals 10774, Cambridge, Massachusetts, USA; 3 Vir Biotechnology, Bellinzona, Switzerland; Chinese Academy of Medical Sciences & Peking Union Medical College, Beijing, China

**Keywords:** elebsiran, tobevibart, HBV, HBsAg, antibody, siRNA, RNAi therapeutic, AAV-HBV mice, human-liver chimeric mice

## Abstract

RNA interference (RNAi) therapeutics targeting hepatitis B virus (HBV) RNAs and monoclonal antibodies (mAbs) targeting HBV surface antigen (HBsAg) represent potential strategies for enabling functional cure in chronic HBV patients. Tobevibart (VIR-3434) is an investigational, Fc-engineered human mAb that targets HBsAg with pan-genotypic neutralizing activity. Elebsiran (VIR-2218) is an investigational small interfering RNA targeting a conserved region of the HBV genome. The *in vitro* antiviral activity of elebsiran was assessed in HBV-infected primary human hepatocytes and hepatoma cells and showed potent inhibition of viral markers HBeAg (EC_50_ of 2.5 nM and 53.7 pM, respectively) and HBsAg (EC_50_ of 1.4 nM and 66.5 pM, respectively). Tobevibart and elebsiran activity *in vivo* was determined using two well-established HBV mouse models: AAV-HBV transduced C57BL/6 mice and human liver-chimeric mice. Mice were treated with a monotherapy or a combination of muHBC34 (the murinized parental mAb of tobevibart) and elebsiran at different doses. In both models, the mouse surrogate of tobevibart or elebsiran monotherapy was effective in reducing blood HBsAg levels. Combined treatment improved suppression of HBsAg (maximum mean reductions of 2.81 log in the AAV-HBV model and 2.51 log in human liver-chimeric mice) and HBV DNA over monotherapy. Tobevibart and elebsiran have been tested in clinical trials for the treatment of chronic hepatitis B and chronic hepatitis Delta.

## INTRODUCTION

Hepatitis B is a viral infection of the liver causing acute and chronic disease. Chronic hepatitis B virus (HBV) infection accounts for approximately 1.1 million deaths per year, mainly due to active liver disease that progresses to cirrhosis and hepatocellular carcinoma (HCC). Although a safe and effective vaccine is used to prevent new infections, no curative treatment is available for the approximately 254 million people living with chronic HBV infection globally (1, 2).

HBV is a member of the *Hepadnaviridae* family of liver-tropic viruses. The virion carries a partially double-stranded DNA genome packaged in a capsid shell and enveloped by a lipid bilayer. Integral to the membrane are the three viral surface proteins: Large, middle, and small HBV surface antigen (HBsAg), which mediate viral entry into hepatocytes via sodium-taurocholate co-transporting polypeptide (NTCP) (3, 4). HBsAg can be assembled in the absence of a viral capsid to form subviral spherical or filamentous particles, which exceed the infectious virions in the serum by 3–6 orders of magnitude in numbers and are thought to be a major contributor to the complex immunopathogenesis of HBV characterized by exhaustion of T- and B-cells (5).

Currently approved treatments for chronic HBV infection include nucleoside/nucleotide analogs (NAs) and pegylated-interferon alpha (PEG-IFNα). NAs bind to the active site of the HBV polymerase and terminate DNA chain elongation, thereby blocking viral replication. NA therapy reduces, but does not eliminate, the risk of HCC and must be administered for life. As NAs target a late step in the viral replication cycle, this therapy does not affect HBsAg expression, resulting in an incidence of HBsAg loss of only about 0.22% per year (6, 7), potentially impacting the immune system’s ability to control the virus. Treatment with PEG-IFNα has broad immunostimulatory properties and can induce HBsAg loss, but only in a small fraction of patients (<10%), and is generally associated with multiple adverse effects (8).

Novel therapies in development aim at achieving functional cure, defined as sustained undetectable levels of HBsAg and HBV DNA after a finite course of treatment (9). Loss of HBsAg is associated with a lower risk of HCC development and minimal risk of relapse after NA treatment discontinuation (10). One strategy to achieve a functional cure is based on the observation that large quantities of HBV antigens, especially HBsAg, contribute to dysfunctional adaptive immune responses. Removing the tolerogen HBsAg from circulation could help restore T- and B- cell responses and control HBV infection (11). Multiple strategies could be employed to decrease HBV antigenemia, including RNA interference (RNAi) to knock-down HBV transcripts and monoclonal antibodies (mAbs) to directly remove HBsAg from circulation.

Elebsiran (VIR-2218) is an investigational small interfering RNA (siRNA) targeting a highly conserved region within the HBx open reading frame (ORF) of the HBV genome, which is part of all major HBV RNA transcripts (12). Elebsiran-mediated RNAi can lead to knock-down of HBV transcripts and reduce HBV antigen production, including HBsAg. Elebsiran contains a conjugated N-acetylgalactosamine (GalNAc) ligand to enable targeted delivery to the liver via the asialoglycoprotein receptor (ASGR1), specifically expressed on hepatocytes. Tobevibart (VIR-3434) is an investigational human mAb targeting the antigenic loop of HBsAg present on virions and SVPs. Tobevibart neutralizes HBV infection with pan-genotypic activity *in vitro,* inhibits viral spread, and decreases HBV viral markers *in vivo (13*). The Fc domain of tobevibart was engineered to increase the binding affinity to Fc gamma receptors (FcgR) IIa and IIIa, while decreasing the affinity to FcgRIIb in order to enhance the clearance of immune complexes through opsonization. Tobevibart and elebsiran have been evaluated in clinical trials in chronic HBV-infected patients, as well as in chronic HBV patients co-infected with hepatitis Delta virus (HDV) (ClinicalTrials.gov identifiers NCT04856085, NCT05461170, NCT06903338, NCT07142811, and NCT07128550).

In this study, we characterized the antiviral activity of tobevibart and elebsiran combination therapy in multiple preclinical HBV models *in vitro* and *in vivo*. We found that combination treatment with both agents led to a potent and sustained reduction of HBV viral markers, including HBsAg.

## RESULTS

### Elebsiran dose-dependently decreases the secretion of HBV antigens *in vitro*

**Fig 1:**
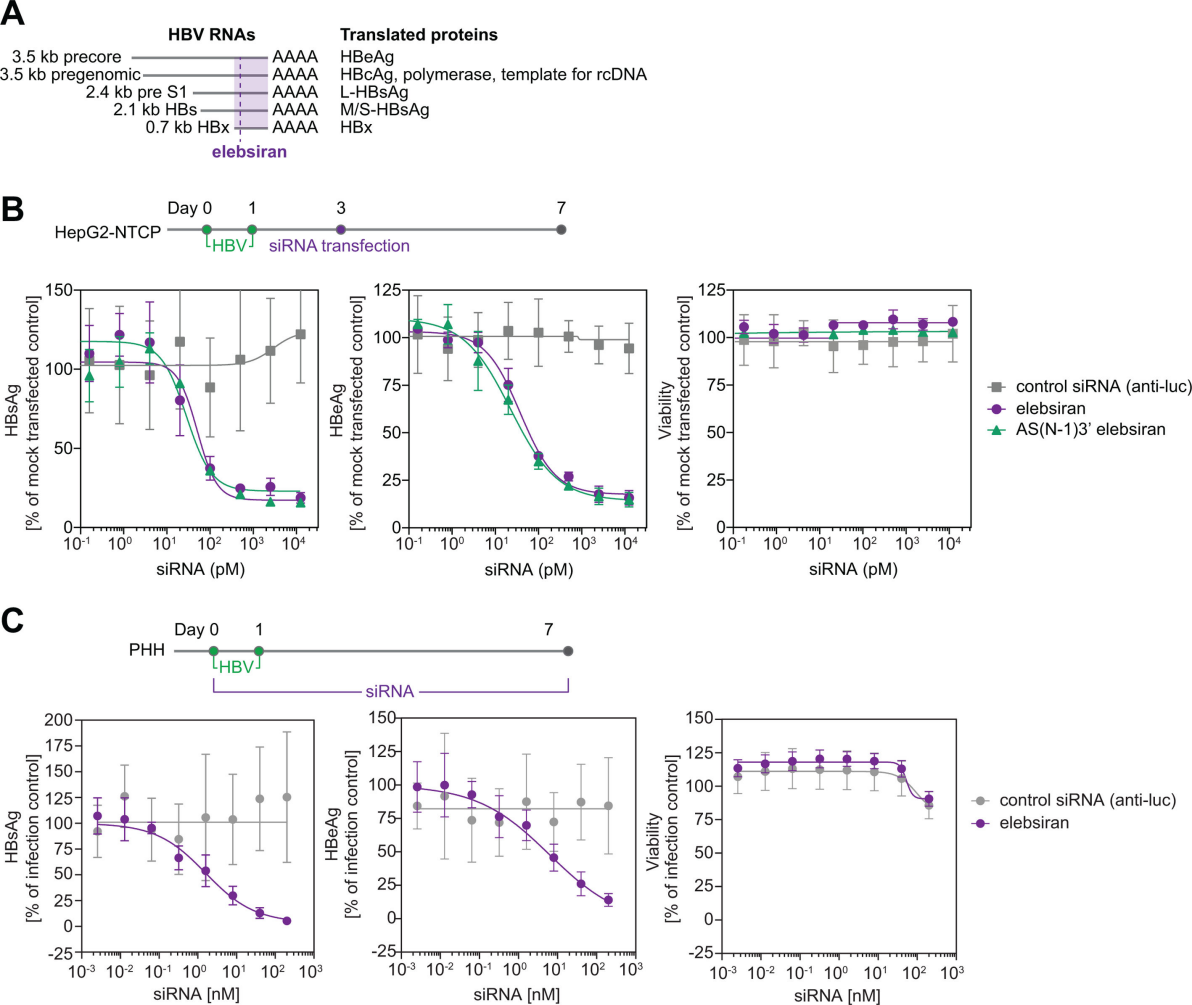
Elebsiran demonstrates antiviral activity against HBV infection *in vitro*. (**A**) Elebsiran targets a region within the HBx gene shared by all HBV RNAs. (**B**) HepG2-NTCP cells were infected with HBV and transfected with siRNA targeting the luciferase gene (control), elebsiran, or AS(N-1)3’ elebsiran at different concentrations 3 days post-infection. HBsAg and HBeAg levels were quantified in the cell supernatant, and cell viability was determined using CellTiter-Glo, 4 days after transfection. Data represent the mean±SD from three technical replicates. Graphs are representative of two independent experiments. (**C**) Serial dilutions of elebsiran or control siRNA were incubated on HBV-infected PHH over 7 days. Secreted viral markers (HBsAg and HBeAg) were quantified, and the cell viability was determined using CellTiter-Glo. Data represent the mean±SD from one of two independent experiments.

We previously confirmed the antiviral activity of tobevibart monotherapy in multiple *in vitro* and *in vivo* models (13). To characterize the *in vitro* activity of elebsiran against HBV, HepG2 cells overexpressing NTCP (HepG2-NTCP) were infected with HBV and transfected 3 days later with elebsiran or a control siRNA. The levels of secreted HBsAg and HBeAg, which are translated from separate HBV RNAs (Fig. 1A), were assessed in parallel. Elebsiran reduced HBsAg and HBeAg levels in a concentration-dependent manner with EC_50_ values of 66.5 pM and 53.7 pM, respectively, without affecting the cell viability (Fig. 1B). Treatment with AS(N-1)3′ elebsiran, a metabolite of elebsiran formed *in vivo* by the loss of one nucleotide from the 3′ end of the antisense strand of elebsiran (14), resulted in highly similar EC_50_ values (44.0 pM for HBsAg and 22.4 pM for HBeAg) (Fig. 1B). GalNAc-dependent uptake of elebsiran in the absence of transfection was confirmed using HBV-infected primary human hepatocytes (PHH). Cells were infected over 7 days in the presence of either elebsiran or a control siRNA in the cell supernatant. Treatment with elebsiran, but not with the control siRNA, led to a concentration-dependent decrease of the two secreted viral markers HBsAg and HBeAg with EC_50_ values of 1.4 nM and 2.5 nM, respectively (Fig. 1C). Importantly, no cytotoxicity was observed upon treatment with either siRNA. Thus, elebsiran shows potent antiviral activity *in vitro*, indicating the efficient suppression of HBV RNAs.

### Elebsiran monotherapy reduces HBsAg levels in the AAV-HBV mouse model.

**Fig 2:**
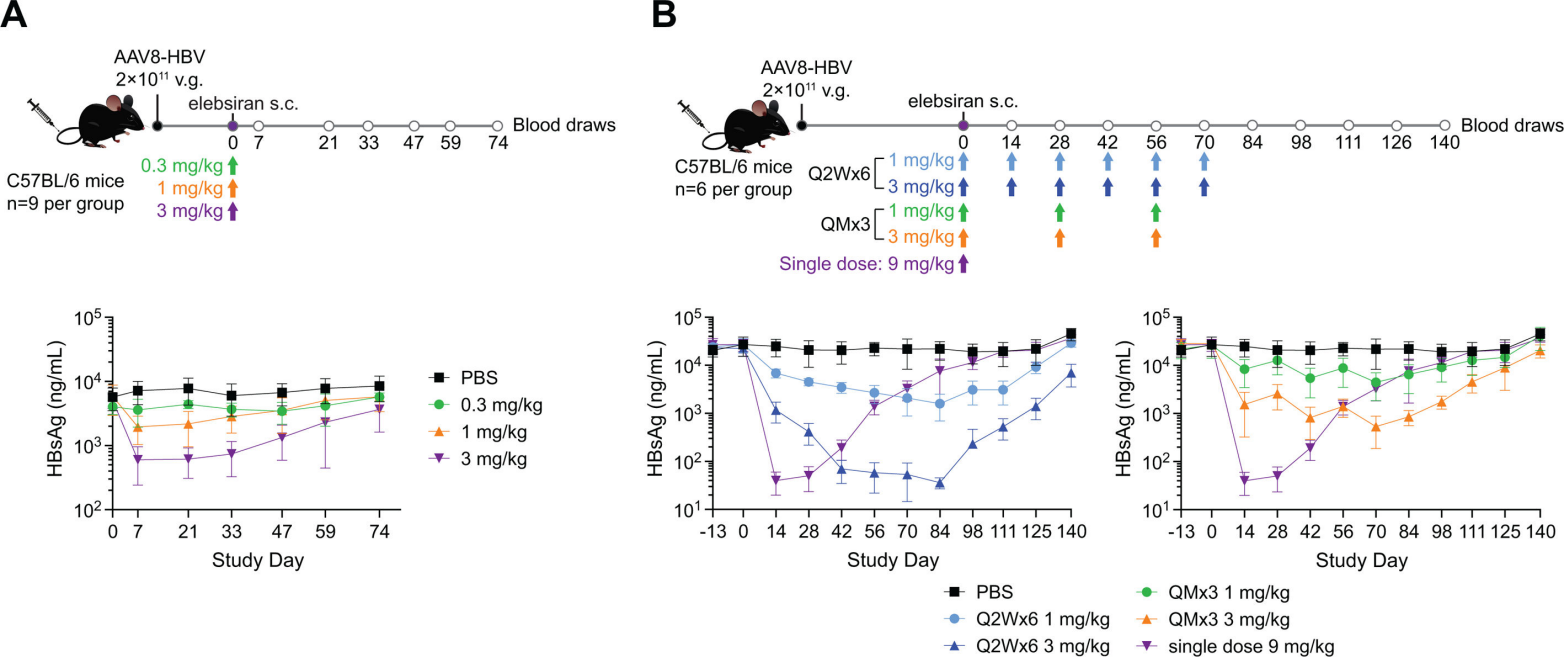
Elebsiran monotherapy reduces HBV viral markers *in vivo* in the AAV-HBV model. (**A**) C57BL/6 mice were injected intravenously via the lateral tail vein with 2 × 10^11^ viral genomes (v.g.) of AAV-HBV/mouse. After a minimum of 2 weeks, a single SC dose of 0.3, 1, or 3 mg/kg elebsiran or phosphate-buffered saline (PBS) was administered. Serum HBsAg levels were assessed at specified time points post-dosing. (**B**) C57BL/6 mice were transduced with AAV-HBV as described in panel **A**. After a minimum of 2 weeks, animals received SC injections of elebsiran at 1 mg/kg or 3 mg/kg either once every 2 weeks for a total of 6 injections (Q2W×6) or once monthly for a total of 3 injections (QM×3) and were compared to mice receiving a single dose of elebsiran at a dose of 9 mg/kg. Serum HBsAg levels were determined at specified time points post-dosing. Each point represents a mean of *N* = 6–9 animals, and the bars represent SD. HBsAg levels are expressed as the percentage relative to pre-dose.

Mice infected with a recombinant adeno-associated virus carrying a replication-competent hepatitis B virus genome (AAV-HBV) establish a persistent HBV replication in hepatocytes with transcription and translation of all HBV ORFs. This preclinical model serves to evaluate antiviral immunity and to assess potential therapeutic approaches for chronic HBV infection (15). To evaluate the *in vivo* activity of elebsiran monotherapy, C57BL/6 mice were transduced with AAV-HBV and approximately 2 weeks later, they were administered with a single subcutaneous (SC) dose of 0.3, 1, or 3 mg/kg elebsiran. Serum HBsAg levels were assessed at specified time points post-dosing, as described in Fig. 2A (upper panel). A single SC injection of elebsiran at a dose range of 0.3 to 3 mg/kg resulted in sustained and dose-dependent reduction in serum HBsAg. The maximum reduction in HBsAg (0.98-log reduction) was observed on day 7 in the highest (3 mg/kg) elebsiran dose group, and this inhibition was maintained through day 33, after which HBsAg levels began rebounding toward baseline (Fig. 2A, lower panel, and Fig. S1A through D).

Next, the antiviral effect of elebsiran was assessed after repeated SC injections in the AAV-HBV mouse model, as described in Fig. 2B (upper panel). Animals received SC injections of elebsiran at 1 mg/kg or 3 mg/kg either once every 2 weeks for a total of 6 injections (Q2W×6) or once monthly for a total of 3 injections (QM×3) and were compared to mice receiving a single dose of elebsiran at a dose of 9 mg/kg.

Animals receiving a single injection of elebsiran at 9 mg/kg had a maximum 2.7-log HBsAg reduction on day 14 and a sustained reduction of serum HBsAg for approximately 4 weeks (Fig. 2B, lower panel, and Fig. S1E and F). Animals that received QM×3 SC injections of elebsiran at 1 or 3 mg/kg exhibited a maximum 0.8- or 1.8-log reduction in serum HBsAg levels on day 70, respectively. The most durable HBsAg decline was observed in animals receiving Q2W×6 SC injections of elebsiran at 1 or 3 mg/kg. This dose regimen resulted in a maximum 1.2- or 2.8-log HBsAg reduction on day 84, respectively. Of note, the 3 mg/kg dose provided a sustained 2-log HBsAg reduction for approximately 8 weeks before serum HBsAg levels rebounded. Taken together, repeated doses of elebsiran demonstrated potent, dose-dependent, and sustained antiviral *in vivo* activity in the AAV-HBV mouse model.

### Combination therapy with elebsiran and muHBC34i reduces HBsAg and HBV DNA plasma levels in AAV-HBV mice

**Fig 3:**
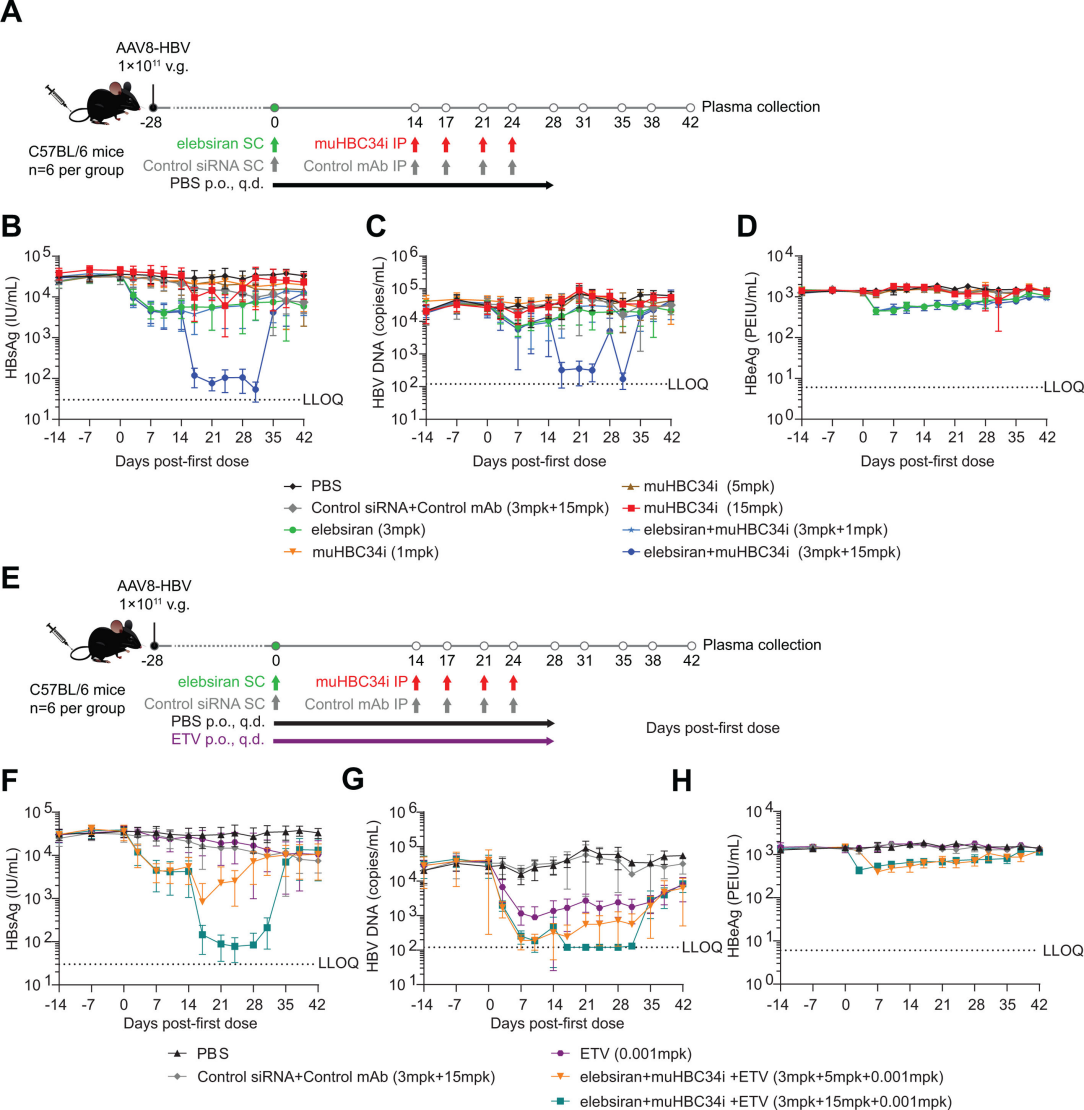
Combination therapy with muHBC34i and elebsiran reduces HBV viral markers in AAV-HBV mice. (**A, E**) Study design. C57BL/6 mice were injected with 1 × 10^11^ v.g. of AAV-HBV (genotype D) in 200 μL of PBS through the tail vein. Starting at 28 days after injection (day 0), treatment was initiated as indicated. Blood was drawn for plasma collection as specified. (**B–H**) Plasma samples were analyzed at the indicated time points for the viral markers HBsAg (**B, F**), HBV DNA (**C, G**), and HBeAg (**D, H**). Each point represents a mean of *n* = 6 animals, and the bars represent SD. Data graphs shown in panels** B–D **and** F–H** are from the same experiment and are split into separate panels for visualization, with identical PBS and Control siRNA+Control mAb data displayed in each panel for reference. SC: subcutaneous, p.o.: by mouth, q.d.: once a day. IP: intraperitoneal, mpk: mg/kg, ETV: entecavir, LLOQ: lower limit of quantification.

Tobevibart is an Fc-engineered, human mAb binding to HBsAg and exerting its antiviral activity by (a) neutralizing viral entry into hepatocytes and (b) binding to HBsAg on virions and SVPs in circulation for opsonization and subsequent removal by immune cells. The latter step requires interaction of the Fc region of the mAb with Fcγ-receptors on immune cells. To evaluate the combination activity of elebsiran and tobevibart versus monotherapy *in vivo*, two murinized versions of tobevibart harboring a mouse IgG2a Fc were generated: HBC34-mu-IgG2a (hereafter abbreviated as muHBC34) is the murinized version of tobevibart containing a mouse IgG2a Fc; HBC34i-mu-IgG2a (hereafter abbreviated as muHBC34i) is an intermediary developability variant of tobevibart in a murinized framework containing a mouse IgG2a Fc. muHBC34 and muHBC34i differ by one amino acid in the light-chain variable region. This difference does not affect HBsAg binding or HBV neutralization activities. C57BL/6 mice transduced with AAV8-HBV (genotype D) were treated with elebsiran, muHBC34i, entecavir (ETV), or a combination of agents at different doses (Fig. 3A and E). Antiviral activity was determined by evaluation of viral plasma markers including HBV DNA, HBsAg, and HBeAg, as well as hepatic HBV DNA levels. In this mouse model, mono-treatment with elebsiran led to a significant reduction in plasma HBsAg, HBeAg, and HBV DNA levels (0.89-log, 0.51-log, and 0.65-log maximum mean reduction, respectively), while muHBC34i monotherapy significantly reduced only plasma HBsAg (0.79-log) and HBeAg (0.19-log) (Fig. 3B through D and Fig. S2A through F). The administration of muHBC34i to elebsiran-pretreated mice further reduced plasma HBsAg and HBV DNA levels when compared to elebsiran monotherapy (2.81-log and 2.44-log maximum mean reduction, respectively). While mice treated with ETV only showed a significant decrease in HBV DNA (1.58-log maximum mean reduction), the triple combination of muHBC34i, elebsiran, and ETV significantly enhanced reductions of plasma HBsAg and HBV DNA (2.62-log and 2.08-log maximum mean reduction, respectively) (Fig. 3F through H and Fig. S2G through I). ETV monotherapy and combination therapy of ETV with elebsiran and both doses of muHBC34i reduced liver HBV DNA significantly on days 28 or 29 but showed no inhibition of liver HBV DNA on days 42 or 43 when ETV had been withdrawn (Fig. S3). Importantly, plasma alanine aminotransferase (ALT) levels remained unaltered in all treatment groups during the experiment when compared to the PBS control group (Fig. S4).

### Combination therapy with muHBC34 and elebsiran reduces HBsAg and HBV DNA serum levels in human liver-chimeric mice

**Fig 4:**
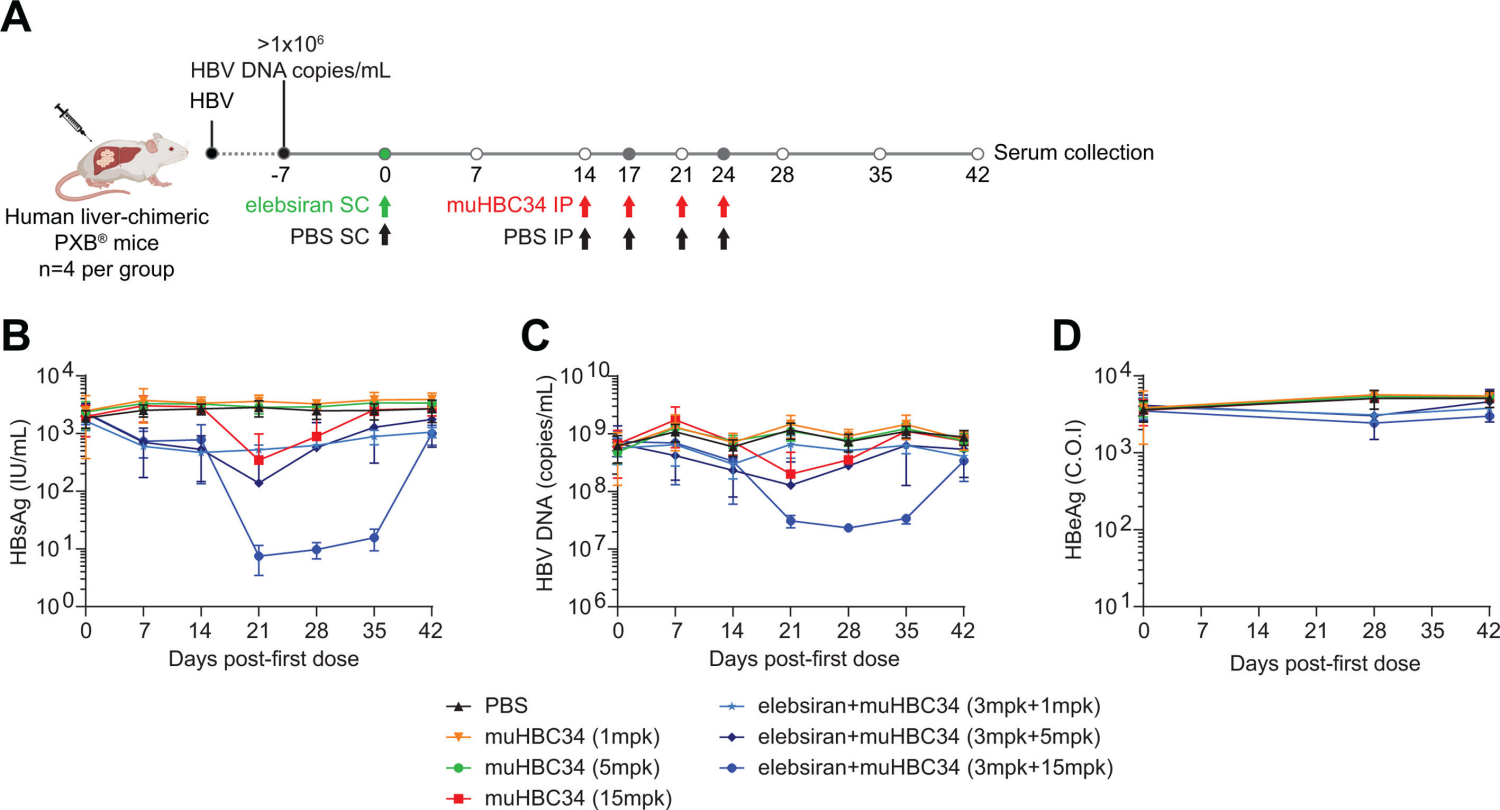
muHBV34 and elebsiran combination therapy reduces HBsAg and HBV DNA serum levels in human liver-chimeric mice. (**A**) Study design. Human liver-chimeric mice (PXB mice) infected with HBV, genotype C, which showed serum HBV DNA levels of 1 × 10^6^ copies/mL or greater 7 days before initiation of the study, were included. On day 0, treatment with the different modalities was initiated as indicated. Blood was drawn for serum collection as specified (empty circles). (**B–D**) Serum samples were analyzed at the indicated time points for the viral markers HBsAg (**B**), HBV DNA (**C**), and HBeAg (**D**). Each point represents a mean of *n* = 4 animals, and the bars represent SD. SC: subcutaneous, IP: intraperitoneal, mpk: mg/kg.

Next, we assessed the antiviral effect of muHBC34 plus elebsiran combination therapy in the human liver-chimeric PXB mouse model. To this end, PXB mice were infected with HBV (genotype C) and treated with either muHBC34 alone or in combination with elebsiran (Fig. 4A). Three different doses of muHBC34 were tested for both the monotherapy and the combination treatment groups, and serum levels of HBsAg, HBeAg, and HBV DNA were assessed at multiple time points post-dosing (Fig. 4A). muHBC34 monotherapy resulted in a substantial but transient decrease in serum HBsAg (1.46-log); the combination of elebsiran and muHBC34 showed an additional ~1-log reduction (total 2.51-log) (Fig. 4B and Fig. S5A and B). The muHBC34 and elebsiran combination also showed a clear reduction in serum HBV DNA levels (1.47-log) when compared to the vehicle group, whereas treatment with muHBC34 alone led to a 0.72-log decrease (Fig. 4C and Fig. S5C and D). No major changes in HBeAg levels were observed in any of the treatment groups (Fig. 4D and Fig. S5E and F).

Taken together, monotherapy with either the mAb muHBC34 or the siRNA elebsiran is effective in reducing plasma/serum levels of HBsAg in both AAV-HBV and liver-chimeric mouse models. Combined treatment provided improved suppression of HBsAg over monotherapy and represents a promising strategy for HBV functional cure.

## DISCUSSION

RNAi is a clinically validated therapeutic approach with eight siRNA therapeutics now FDA-approved to date, all of which are liver-specific and target host genes for indications including transthyretin-mediated amyloidosis, hypercholesterolemia, and primary hyperoxaluria (16, 17). While siRNAs have historically been used in hereditary diseases, multiple siRNA candidates are currently in preclinical and clinical development for infectious diseases. With the advancements of targeting technologies to extrahepatic tissues, especially targeting to the lung via inhalation (18), RNAi therapeutics have shown promise for SARS-CoV-2 (19, 20), influenza (21), HIV (22), and RSV (23). HBV replicates exclusively in hepatocytes that can be targeted via GalNAc-based chemical modifications on the siRNA, which is a well-established concept for liver-specific delivery (24). Due to the 3′ overlapping nature of the HBV RNAs, a single siRNA targeting the 3′ region (within the HBx ORF) has the potential to knock-down all viral transcripts. First-generation siRNAs targeting the DR1 region have failed to knock-down HBV transcripts derived from genomic integrants, due to the genomic breakpoints that occur during double-stranded linear DNA formation in the step prior to genomic integration (25). Elebsiran has been designed to target the HBx ORF region (12), well upstream of the DR1 site to ensure knock-down of cccDNA-derived and integrant-derived viral RNAs.

Novel therapies in development for chronic HBV infection aim at achieving functional cure, defined as durable HBsAg and HBV DNA loss after cessation of therapy. Loss of HBsAg is associated with a favorable prognosis, but spontaneous seroclearance only occurs in about 1%–2% of cases per year (26). HBsAg is produced in the infected hepatocyte from either viral cccDNA or integrated HBV DNA and secreted into circulation, leading to vast amounts of circulating HBsAg up to >10,000 IU/mL. HBsAg plays a dual role in the viral replication cycle: (i) it is an essential envelope protein, required for viral assembly, secretion, and entry into hepatocytes (3); (ii) it contributes to the immune dysfunction in chronic HBV infection by acting as an immune decoy to mask endogenous anti-HBsAg antibodies and promote the exhaustion and dysfunction of virus-specific T- and B-cells (11). High levels of serum HBsAg have been directly correlated with the magnitude of immune cell dysfunction and inhibitory receptor expression (27).

Here, we provide two strategies to lower serum HBsAg using RNAi and an mAb targeting HBsAg. While monotherapy with tobevibart or elebsiran in AAV-HBV and liver-chimeric mouse models of HBV significantly reduced serum levels of HBsAg (AAV-HBV mice: 0.79–0.89-log; liver-chimeric mice: 1.46-log), maximum reduction was achieved when combining both agents (2.81-log in AAV-HBV mice and 2.51-log in liver-chimeric mice). This modest reduction induced by tobevibart monotherapy is potentially due to the high baseline levels of HBsAg (>4-log10 and >3-log10, respectively), as opsonization and clearance of SVPs by tobevibart occurs in a stoichiometric way and bound mAb is removed by target-mediated drug disposition. Conversely, knock-down of HBV RNAs by elebsiran and the consequent reduction in HBsAg production lowered serum HBsAg to below a threshold where the elimination of HBsAg mediated by tobevibart was more pronounced.

While HBV-targeting RNAi therapeutics mainly exert their antiviral activity via knock-down of viral antigen production including HBsAg and HBeAg, one additional mode of action potentially stems from reducing the levels of the smallest HBV transcript, encoding for HBx. HBx has been shown to interfere with multiple cellular pathways and is essential for transcription of cccDNA, mechanistically via degradation of the host proteins SMC5/6 that naturally restrict cccDNA accessibility (28, 29). In a recent study, Allweiss et al. treated HBV-infected liver-chimeric mice with siRNAs targeting the HBx region and observed a reduction in intra-liver HBx protein levels, associated with reappearance of SMC5/6 expression and decreased intracellular HBV RNA levels. Whether this epigenetic suppression of HBV cccDNA is also induced by treatment with elebsiran remains to be investigated (30).

In addition to treating chronic HBV, tobevibart and elebsiran are being studied for treating chronic HDV infection, the most severe form of viral hepatitis (31). Since HDV requires HBsAg for assembly and spread, agents targeting HBsAg can affect HDV infection (13, 32, 33). We recently reported that tobevibart and elebsiran, both as monotherapy and combination therapy, potently reduced viral markers in HBV-/HDV-coinfected liver-chimeric mice, and, similar to our current study, the strongest decrease in viral markers was achieved with the combination therapy (32). As NRTIs have no effect on HDV viral load (34), a potent and durable reduction of HDV RNA in the serum, as seen in mice treated with tobevibart and elebsiran (31), could have important clinical significance through decreasing HDV-infected cells and associated liver inflammation.

In conclusion, tobevibart and elebsiran are two novel highly potent investigational agents that exert their antiviral activity via multiple complementary mechanisms: (a) downmodulation of HBV RNAs and reduction in HBV antigen production, (b) inhibition of HBV entry into hepatocytes, and (c) reduction of circulating HBsAg by direct opsonization and clearance of SVPs and virions. Consequently, the combination of tobevibart and elebsiran has entered phase III trials for the treatment of HDV. Further evaluation would be necessary to determine whether the combination can induce functional cure in patients with HBV in combination with other immunomodulatory agents.

## MATERIALS AND METHODS

### Cell culture

HepG2 cells (ATCC, cat. CRL-10741) and HepAD38 cells (provided by Christoph Seeger, Fox Chase Cancer Center, Philadelphia) were cultured in DMEM with 10% FBS and 1× penicillin-streptomycin. HepG2 cells stably overexpressing NTCP (HepG2-NTCP) were generated via lentivirus transduction and selected using puromycin (13). For infection, 2% DMSO was added to the culture medium. PHH (Thermo Fisher Scientific, cat. # HMCPTS) were thawed and plated in Williams E medium with primary hepatocyte thawing and plating supplements, 2% FBS, and 2% DMSO and maintained in Williams E medium with primary hepatocyte maintenance supplements, 2% FBS, and 2% DMSO. All cells were grown at 37°C and 5% CO_2_. Cell lines tested negative for mycoplasma contamination.

### HBV production

Virus was purified from cell culture supernatants from HepAD38 cells, which express HBV (genotype D) under the control of a tetracycline-responsive promoter. Viral particles were precipitated with 6% PEG-8000 (Sigma-Aldrich) overnight at 4°C, followed by centrifugation and resuspension in 20 mM Tris/140 mM NaCl buffer containing 10% FBS. Aliquots of the virus stock were stored at −80°C.

### Reagents

Elebsiran (VIR-2218), the metabolite AS(N-1)3′ elebsiran, and the anti-luciferase control siRNA were produced by Alnylam Pharmaceuticals (Boston, USA). HBC34-mu-IgG2a (hereafter abbreviated as muHBC34) is the murinized version of tobevibart containing a mouse IgG2a Fc. HBC34i-mu-IgG2a (hereafter abbreviated asmuHBC34i) is an intermediary developability variant of tobevibart in a murinized framework containing a mouse IgG2a Fc. muHBC34 and muHBC34i differ by one amino acid in the light-chain variable region. This difference does not affect HBsAg binding or HBV neutralization activities. Both antibodies were produced in-house at Vir Biotechnology, Inc. (Bellinzona, Switzerland).

### HepG2-NTCP infection and siRNA treatment

HepG2-NTCP cells were seeded at 35,000 cells/well in a 96-well plate. The next day (Day 0), the infection mix was prepared by diluting the concentrated HBV stock virus prepared from HepAD38 cells to 370 GE/cell in infection medium (DMEM, 10% FBS, 1× penicillin-streptomycin, 2% DMSO) with 4% PEG8000. The medium was removed from the cells, and 100 μL infection mix was added per well. For the 100% inhibition control wells, cells were preincubated for 30 min with 1 μM Myrcludex B (MyrB, synthesized by GenScript, USA), and the same concentration of MyrB was additionally added into the infection mix for these wells. One day post-infection, the inoculum was removed, cells were washed with PBS, and fresh infection medium was added. MyrB was added at the same concentration to the 100% inhibition control wells during the infection. On day 3, cells were transfected with test and control articles using the Lipofectamine RNAiMAX transfection reagent. Five-fold, eight-step serial dilutions of AS(N-1)3′ VIR-2218, VIR-2218, and anti-luciferase siRNA were prepared in OptiMEM starting at 12,500 pM (from 12,500 pM to 0.16 pM). Mock-transfected control wells contained OptiMEM mixed with PBS alone. Lipofectamine RNAiMAX reagent was diluted in OptiMEM in a separate tube, and an equal volume of diluted Lipofectamine RNAiMAX reagent was mixed with the diluted siRNA. After incubation for 5 min at room temperature (RT), 10 μL transfection mix was dispensed dropwise in each well. On day 4, the transfection reagent was removed, cells were washed with PBS, and fresh infection medium was added. The medium was exchanged and MyrB was replenished every 2–3 days post-transfection. On day 10, culture supernatants were collected and analyzed for HBsAg and HBeAg using CLIA, and the cells were analyzed for viability with CellTiter-Glo.

### Free siRNA uptake assay on PHHs

PHHs were seeded at 50,000 cells/well in collagen-coated 96-well plates according to the manufacturer’s instructions. Five h post-seeding, at ~90% confluence, infection was initiated. Infection mix was prepared by diluting the concentrated HBV stock virus at 1:33 in PHH maintenance medium containing 4% PEG8000. Five-fold, eight-step serial dilutions of the test article elebsiran or a luciferase-targeting siRNA (control) were prepared in PBS starting at 2 μM and diluted 1:10 into the infection mix. Infection control wells contained infection mix with PBS alone. The medium was removed from the cells, and 100 μL infection mix was added per well. One day post-infection, the inoculum was removed, cells were washed with PBS, and fresh medium with freshly prepared test article dilutions at the same concentrations as during infection was added. The medium was exchanged, and test articles were renewed every 2–3 days post-infection. At day 7 post-infection, the cell supernatant was used for HBeAg/HBsAg CLIA, and the cells were analyzed for viability with CellTiter-Glo.

### HBsAg and HBeAg CLIA from cell culture supernatants

HBeAg and HBsAg chemiluminescence immunoassay kits were used following the manufacturer’s instructions for the quantitative detection of HBeAg and HBsAg, respectively (Autobio Diagnostics). The standard curve and buffers provided in the kit were used, unless otherwise noted. Cell culture supernatants from infected cells were centrifuged at 1,500 rpm to remove cellular debris, diluted 1:4 in PBS, and 50 μL of each sample dilution was pipetted into each well of the antibody-coated microtiter plate. Standard curves were pipetted in duplicate. Then, 50 μL of the enzyme-conjugated detection antibody were added to each well, and the plates were shaken for 60 s on a plate shaker at 150 rpm prior to incubation at 37°C for 1 h. The plates were decanted, blotted on adsorbent paper, and washed 6 times with washing buffer. Then, 50 μL of premixed substrate was added, and the plates were incubated in the dark for 10 min. The luminescence signal was acquired using a SpectraMax iD5 plate reader (Molecular Devices) using the following settings: luminescence, endpoint measurement, opaque plate, columns read, 1 s read time, and 1 mm read height.

### Cell viability determination

The CellTiter-Glo luminescent cell viability assay was used for the quantitative detection of cell viability according to the manufacturer’s recommendations. CellTiter-Glo reagent was diluted with an equal volume of PBS. The cell culture supernatant was removed, and 100 uL of the diluted CellTiter-Glo reagent was pipetted into each well. The plates were incubated in the dark with 300 rpm shaking for 10 min. Sample solutions were transferred into 96-well black plates. The luminescence signal was acquired on a EnSight multi-mode plate reader (PerkinElmer) using the following settings: luminescence, single well, 0.1 mm read height, and 0.1 s measurement time.

### Elebsiran treatment of AAV-HBV mice

Mouse studies were conducted at Alnylam Pharmaceuticals Inc. (Boston, USA). AAV8-HBV from SignaGen Laboratories (Catalog No. SL00862) was diluted in 1× PBS (Gibco, Cat#20012-027) to a final concentration of 2 × 10^12^ genome copies (GC)/mL. Male C57BL/6 mice 6–8 weeks of age from Charles River Laboratories were injected intravenously via the lateral tail vein with 2 × 10^11^ GC/mouse in a fixed volume of 100 μL. Before initiating siRNA studies, a minimum of 2 weeks was allowed to pass following the AAV8-HBV transduction to ensure stable HBV expression in the mice. Elebsiran was diluted with sterile 1× PBS and administered with a variable volume of 10 µL/g.

For single dosing experiments, animals (*n* = 9 in each treatment group) received a single SC dose of PBS or elebsiran at 0.3, 1, or 3 mg/kg. Blood was collected at Days −24,−2, 0, 14, 21, 33, 47, 59, and 74 postdose via retro-orbital sinus into serum separator tubes and allowed to clot for 30 min. For multiple dosing experiments, each dose group consisted of 6 animals per dose group. Animals in Groups 1 to 3 received SC injections of PBS or elebsiran (1 mg/kg) Q2W for a total of 6 injections. Animals in Group 4 received SC injections of elebsiran at 1 mg/kg QM for a total of 3 injections. Animals in Group 5 received SC injections of elebsiran at 3 mg/kg Q2W for a total of 6 injections. Animals in Group 6 received SC injections of elebsiran at 3 mg/kg QM for a total of 3 injections. Animals in Group 7 received a single SC injection of elebsiran at 9 mg/kg. Blood was collected at Days −55,−27, −13, 0, 14, 28, 42, 56, 70, 84, 98, 111, 126, and 140 postdose via retro-orbital sinus into serum separator tubes and allowed to clot for 30 min. Blood was spun in a microcentrifuge for 10 min at 13,000 rpm and 4°C. Serum was aspirated and stored at −20°C.

For the single dosing studies, HBsAg protein levels were evaluated using an ELISA kit from BioTang, Lexington, MA 02421 (Catalog No. HU9455). The US Biologics (Memphis, TN) HBsAg protein (Catalog No. H1910-27) was used to generate the standard curve. Serum samples were diluted in 1× PBS from Gibco, Gaithersburg, MD (Catalog No. 14190-144) at 1:2,000 or 1:500 and analyzed for HBsAg according to the manufacturer’s protocol with minor modifications. Briefly, 50 μL/well of diluted serum or standard were loaded in duplicate into the plate and incubated for 1 h at 37°C. After this incubation, 50 μL of the enzyme conjugate was added to each well, and the plate was incubated at 37°C for 30 min. The plate was washed 3 times with 300 μL/well 1× wash buffer, then blotted until all of the liquid was removed from the wells, 100 μL/well substrate was added, and plates were incubated at 37°C for 30 min. Finally, an additional 100 μL/well of stop solution was added, and the absorbance was measured at 450 nm. Where the HBsAg level fell below the lower limit of quantification (LLOQ) of the assay (313 ng/mL), values were recorded as below the limit of quantification (BLQ). For the generation of graphs, the LLOQ (313 ng/mL) was assigned for all BLQ results.

For the multiple dosing experiments, HBsAg protein levels were analyzed using an ELISA kit from BioRad Laboratories, Hercules, CA (Catalog No. 32591), and the BioRad recombinant HBsAg (AY) protein (Catalog No. OBT0915US) was used to generate the standard curve. Serum samples were diluted 1:12,000 through 1:100 in 1× DPBS (Gibco, Catalog No. 14190-144), and HBsAg levels were evaluated using the ELISA protocol A per manufacturer’s instructions. Briefly, 100 μL/well of diluted serum or standard were loaded into the plate and incubated for 1 h at 37°C. After this incubation, plates were washed 5 times with wash buffer including a >45 -s soak between each wash and then blotted until all of the liquid was removed from the wells. The conjugate (100 μL) was added to each well, and the plate was incubated for 1 h at 37°C. Plates were washed 5 times with wash buffer including a >45 -s soak between each wash and then blotted until all of the liquid was removed from the wells. The substrate (100 μL) was added to each well, and plates were incubated in the dark at RT for 30 min. Finally, an additional 100 μL/well of stop solution was added, and the absorbance was measured at a wavelength of 450 nm with a correction of 615 nm. Serum collected on Day 0 was included in each ELISA run and used to calculate HBsAg reduction relative to each ELISA run. For samples where the HBsAg level was below the detection range of the assay, results are reported as BLQ. For the generation of graphs, the concentration of the lowest detectable standard corrected for sample dilution was assigned to all BLQ results (LLOQ, 31.3 ng/mL).

### Elebsiran and muHBC34i treatment of AAV-HBV mice

Mouse study was conducted at Wuxi AppTec (Shanghai, China). Male, 5-week-old, specific pathogen-free C57BL/6 mice were obtained from SLAC (Shanghai SLAC Laboratory Animal Co., Ltd.). The mice were housed in the animal care facility in individually ventilated cages after the animals arrived at the WuXi animal facility. The study was approved by the WuXi IACUC (Institutional Animal Care and Use Committee, IACUC protocol N20160810-Mouse). On day −28, mice were injected with 1 × 10^11^ genome equivalents of AAV-HBV (rAAV8-1.3HBV, genotype D, Beijing FivePlus Molecular Medicine Institute) in 200 μL of PBS through the tail vein. On days −14 and −7, all the AAV-/HBV-inoculated mice were bled (~100 µL blood/mouse) via submandibular vein puncture for plasma preparation. Blood samples were collected using K2-EDTA as the anti-coagulant, centrifuged at °C, 7,000 g for 10 min for plasma collection. The plasma samples were transferred to the *in vitro* group for the detection of HBV DNA, HBsAg, and HBeAg for viral infection level determination for grouping. On day 0, 84 mice with qualified viral infection levels were selected and randomly grouped for administration of the test articles (*n* = 6 per group). There were no statistically significant differences between groups in HBV DNA, HBsAg, and HBeAg, as well as body weight. The mice were dosed with vehicle or test articles according to the treatment regimens. On days 0 (pre-doing), 3, 7, 10, 14, 17, 21, 24, 28, 31, 35, and 38, all the treated mice were bled via submandibular vein puncture for plasma collection for detection including HBV DNA, HBsAg, HBeAg, and ALT. On days 28/29, three mice per group, and on day 42/43, another 3 mice per group were sacrificed for blood and tissue collection. HBV DNA in plasma and liver was quantified by an in-house qPCR assay. HBeAg in mice plasma was detected using the HBeAg ELISA kit (Autobio, CL 0312) following the manual. HBsAg in mice plasma was determined using Architect i2000 by a third-party partner (KingMed Diagnostics). ALT levels in plasma were detected using the ALT Activity Assay Kit (Sigma, MAK052) following the manufacturer’s manual. Statistical analysis was performed using Student’s t-test.

### Elebsiran and muHBC34 treatment of liver-chimeric PXB mice

Mouse study was conducted at PhoenixBio Co., Ltd (Hiroshima, Japan). PXB-mice with the genotype cDNA-uPA^wild/+^/SCID (urokinase-type plasminogen activator/severe combined immunodeficiency) and transplanted with human hepatocytes with an estimated replacement index of 70% or more based on human albumin (hAlb) concentration were used. Male PXB-mice (23–26 weeks old) were infected with HBV genotype C until serum HBV DNA reached a plateau of >1 × 10^6^ copies/mL, 7 days prior to the start of the study. Mice were randomized based on body weight, serum HBV DNA levels, and blood hAlb concentration into the indicated treatment groups each containing 4 mice. Elebsiran was administered subcutaneously (s.c.) as a single dose (3 mg/kg) on day 0 and muHBC34 intraperitoneally (i.p.) as multiple doses (each 1, 5, or 15 mg/kg) on days 14, 17, 21, and 24. Saline was used as a vehicle control and administered s.c. on day 0 and i.p. on days 14, 17, 21, and 24. Whole blood was collected from all animals via the tail vein at each time point (pre-dosing) on days 0, 7, 14, 21, 28, 35, and 42. Serum was obtained via coagulation (1 h at RT) and centrifugation (4°C, 11,000 × *g*, 10 min). On Day 42, all surviving animals were anesthetized with isoflurane, and a minimum of 400 μL of blood was collected from each animal via the heart into syringes, after which the animals were sacrificed by cardiac puncture and exsanguination.

#### Blood hAlb measurement

Two microliters of blood were diluted in saline. The clinical chemistry analyzer (BioMajestyTM Series JCABM6050, JEOL Ltd., Tokyo, Japan) was used to measure the blood hAlb concentration using latex agglutination immunonephelometry (LZ Test “Eiken” U-ALB, Eiken Chemical Co., Ltd., Tokyo, Japan).

#### Serum HBV DNA quantification

HBV DNA was extracted from 5 μL of serum using the SMITEST EX-R&D Nucleic Acid Extraction Kit (MEDICAL & BIOLOGICAL LABORATORIES CO., LTD., Nagoya, Japan). The DNA was dissolved in 20 μL nuclease-free water (Ambion). The real-time detection PCR to measure the serum HBV DNA concentration was performed using the TaqMan Fast Advanced Master Mix (Applied Biosystems, Thermo Fisher Scientific Inc.) and ABI Prism 7500 sequence detector system (Applied Biosystems). The PCR mixture was added into 5 μL of the extracted DNA. The initial activation of uracil-N-glycosylase at 50°C for 2 min was followed by polymerase activation at 95°C for 20 s. Subsequent PCR amplification consisted of 53 cycles of denaturation at 95°C for 3 s and annealing and extension at 60°C for 32 s per cycle in an ABI 7500 sequence detector. The average serum HBV DNA level was calculated from the values of the two separate wells. The primers and probes consisted of the following: forward primer (5′-CACATCAGGATTCCTAGGACC-3′), reverse primer (5′- AGGTTGGTGAGTGATTGGAG-3′), and TaqMan probe (6-FAM- CAGAGTCTAGACTCGTGGTGGACTTC-TAMRA). The LLOQ of this assay was 4.0 × 10^4^ copies/mL serum.

#### Serum HBsAg quantification

Serum HBsAg concentration was determined by SRL, Inc. (Tokyo, Japan) based on Chemiluminescent Enzyme Immuno Assay (CLEIA) developed by Fujirebio, Inc. (LUMIPULSE HBsAg-HQ, LUMIPULSE Presto II). The dilution factor was 30, and the measurement range of this assay was between 0.005 and 150.000 IU/mL. For the 30-fold diluted samples, the measurement range was adjusted to be between 0.15 and 4,500.000 IU/mL.

#### Serum HBeAg quantification

Serum HBeAg concentration was determined by SRL, Inc. based on CLEIA developed by Fujirebio (LUMIPULSE HBeAg, LUMIPULSE Presto II). The dilution factor was 30, and the measurement range of this assay was between 0.1 and 1,590 C.O.I. For the 30-fold diluted samples, the measurement range was adjusted to be between 3 and 47,700 C.O.I.

## SUPPLEMENTAL MATERIAL

Supplemental figures aac.01127-25-s0001.pdf Fig. S1 to S5.

ASM does not own the copyrights to Supplemental Material that may be linked to, or accessed through, an article. The authors have granted ASM a non-exclusive, world-wide license to publish the Supplemental Material files. Please contact the corresponding author directly for reuse.
