# Supplementary material for: Combination therapy with tobevibart and elebsiran potently reduces hepatitis B virus surface antigen levels in preclinical in vivo models
Source: Antimicrob Agents Chemother. 2026 Jan 26;70(3):e01127-25. doi: 10.1128/aac.01127-25 (PMC12959159; doi:10.1128/aac.01127-25)
Supplement: Supplemental figures — Fig. S1 to S5. [file aac.01127-25-s0001.pdf]

Supplemental Figures

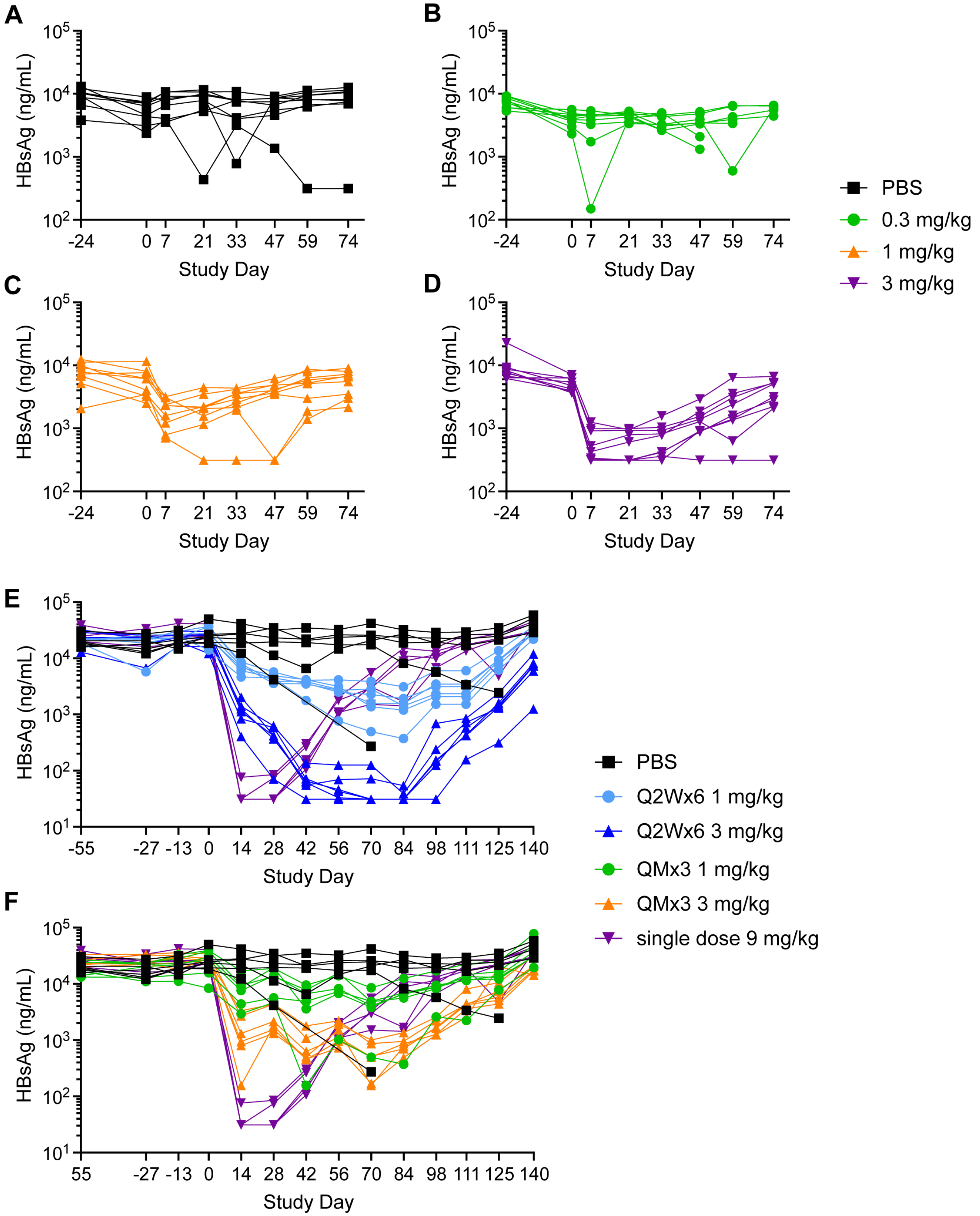

Figure S1: Serum HBsAg levels in individual mice. Related to main Fig. 2A (A-D) and Fig. 2B (E-F).

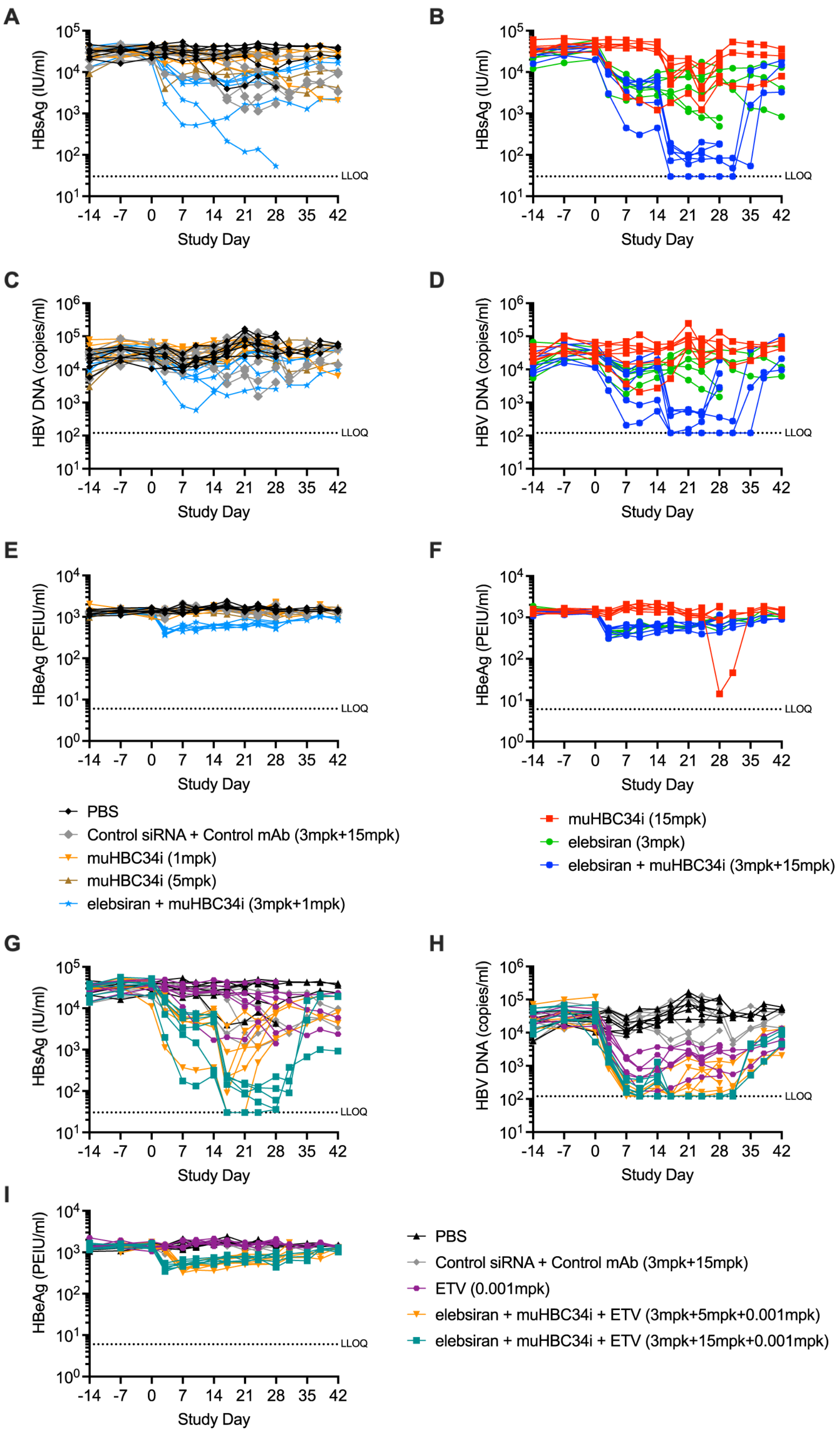

Figure S2: Plasma HBsAg, HBV DNA and HBeAg levels in individual mice. Related to main Figs. 3A-D (A-F) and Figs. 3E-H (G-I).

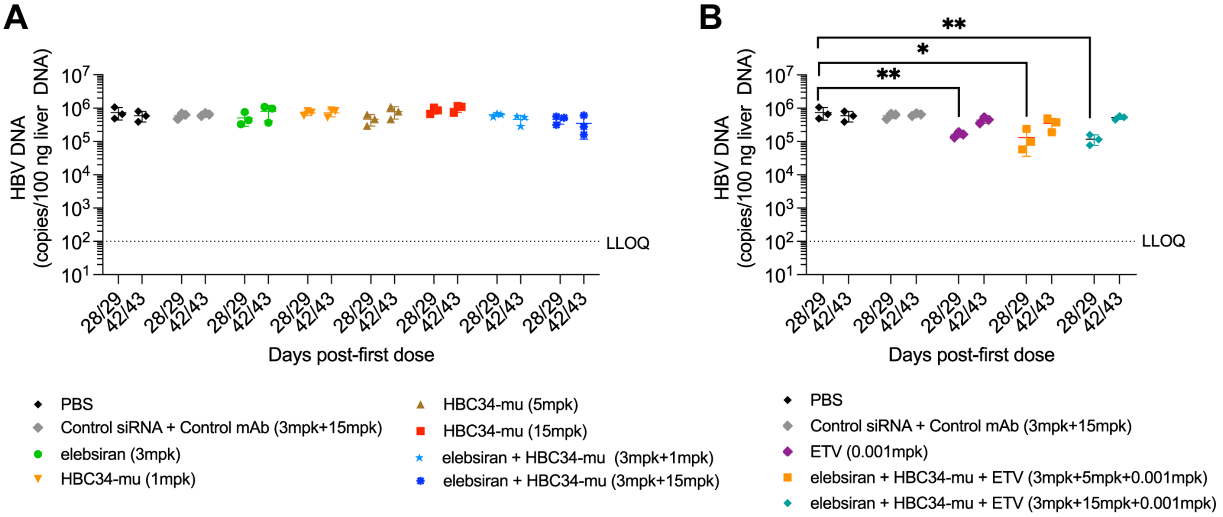

Figure S3: HBV DNA levels in mouse liver. HBV DNA levels in the liver of mice corresponding to the study shown in Figure 3 was determined by qPCR at the indicated time-points. The error bar represents SD.

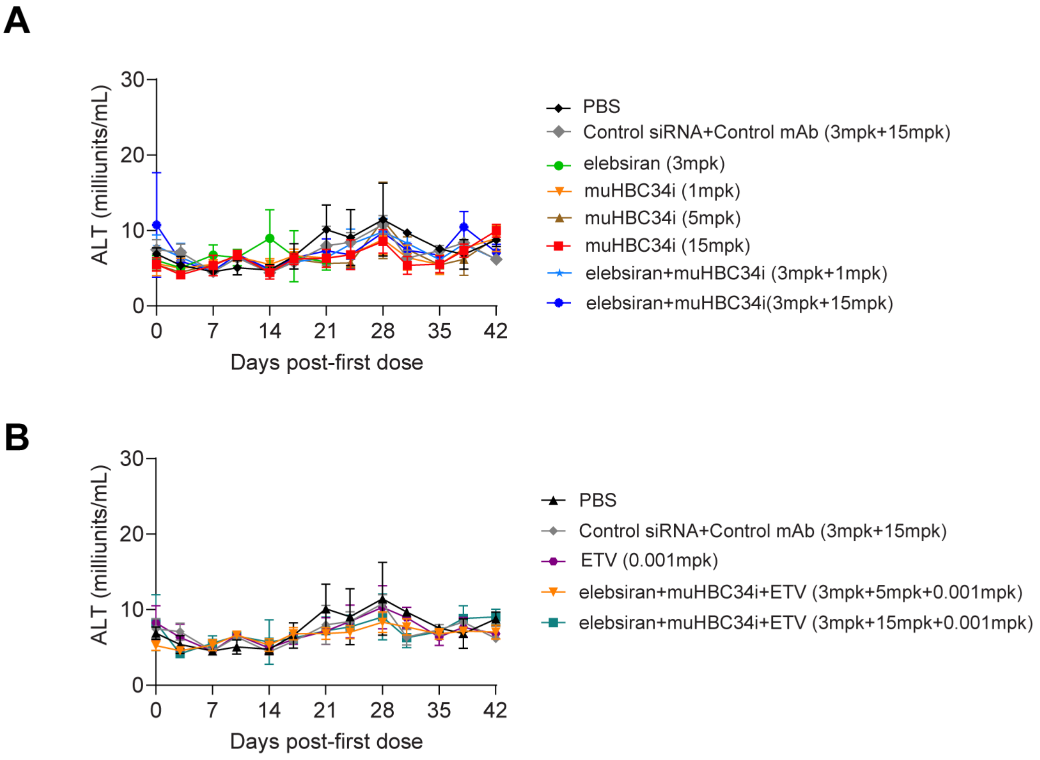

Figure S4: Plasma ALT in AAV-HBV mice. ALT in plasma in mice corresponding to the study shown in Figure 3 was determined with the ALT Activity Assay Kit. The error bar represents SD.

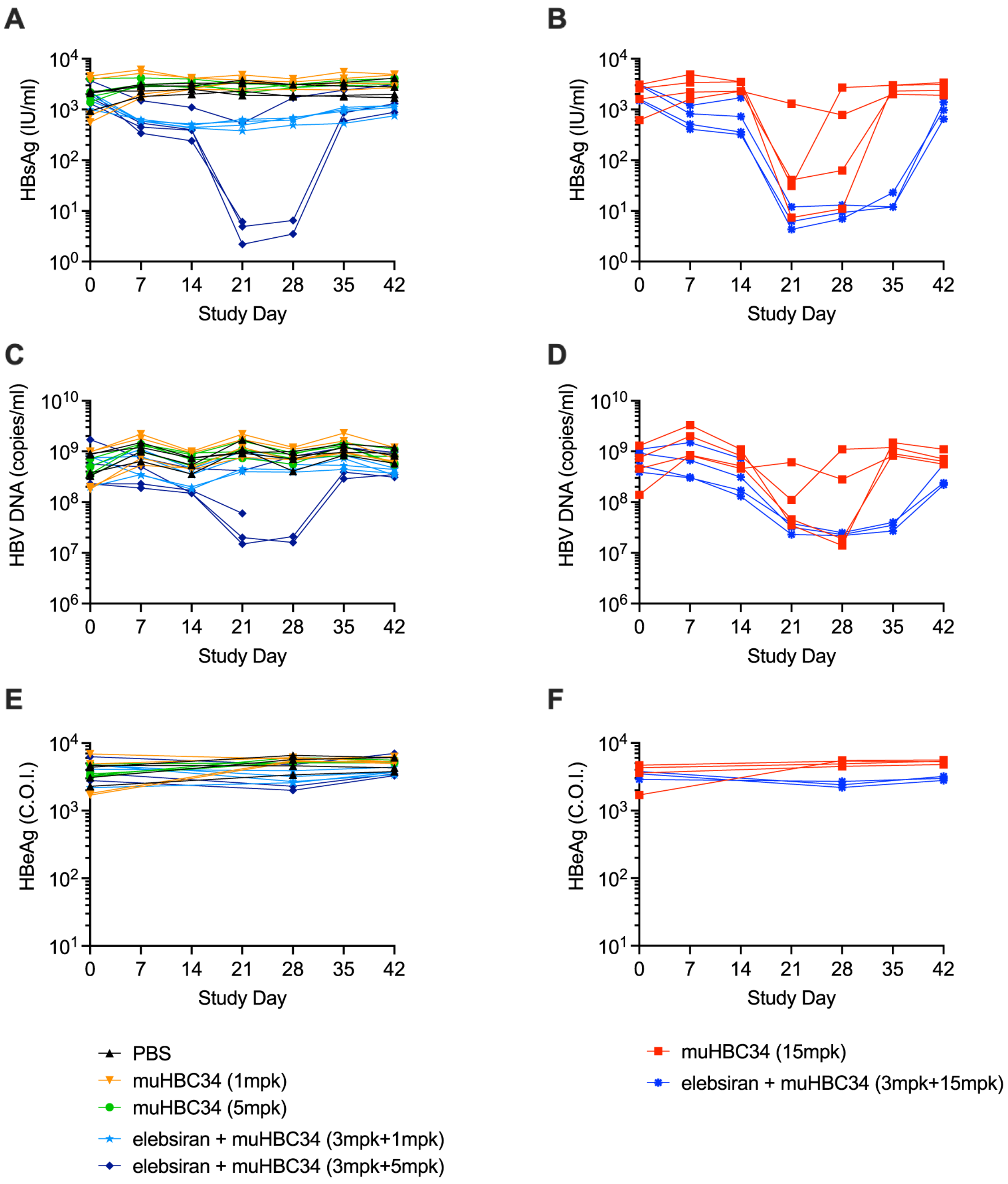

Figure S5: Serum HBsAg, HBV DNA and HBeAg levels in individual mice. Related to main Figs. 4B (A-B), 4C (C-D) and 4D (E-F).
